# A Systematic Review of a Long-forgotten Cause of Atrial Fibrillation and Stroke: Cor Triatriatum

**DOI:** 10.7759/cureus.6371

**Published:** 2019-12-13

**Authors:** Waqas Ullah, Yasar Sattar, Hiba Rauf, Sohaib Roomi, Murtaza ishaq Shah

**Affiliations:** 1 Internal Medicine, Abington Hospital - Jefferson Health, Abington, USA; 2 Internal Medicine, Icahn School of Medicine at Mount Sinai, New York, USA; 3 Internal Medicine, Dow Medical College, Karachi, PAK; 4 Internal Medicine, Services Institute of Medical Sciences, Lahore, PAK

**Keywords:** cor triatriatum, cor triatriatum sinister, cor triatriatum dexter, tri atrial, atrial fibrillation, stroke

## Abstract

Cor triatriatum is a rare congenital cardiac condition characterized by the division of one atrium into two chambers by a fibromuscular membrane, resulting in three atrial chambers. The goal of this study was to determine the associations of cor triatriatum with cyanosis, atrial fibrillation (AF), and stroke.

MEDLINE (PubMed, Ovid), Embase, and Cochrane databases were searched on April 25, 2019, for relevant articles on cor triatriatum. After initial screening and removal of duplicates, 235 articles were selected. Data were extracted from these articles, including types, presentations, diagnostic findings, management, and outcomes of patients with cor triatriatum.

Approximately 83% of patients with cor triatriatum had cor triatriatum sinistrum (CTS) and 17% had cor triatriatum dextrum (CTD). The mean age of all patients was 29±23 years. Mean ages at diagnosis differed significantly in patients with CTS and CTD (31±23 years vs. 21±20 years, p=0.02). CTS showed a significantly greater association with AF (14.65% vs. 12.5%, p=0.036) and had a substantially higher risk of stroke (7.9% vs. 5.0%, p=0.04) than CTD. CTS also had a numerically higher association with atrial septal defects (15.13% vs. 15.6%), but this difference was not statistically significant (p=0.89). In contrast, cyanosis at presentation was significantly more frequent in patients with CTD than CTS (5.5% vs. 5.3%, p=0.05). Management did not differ significantly between these groups (p=0.29). The overall mortality rate was 16%, with no significant difference between patients with CTS and CTD (p=0.33).

The higher likelihood of AF and stroke in CTS than in CTD patients warrants treatment of the former with anticoagulation agents, irrespective of their CHA₂DS₂-VASc scores (congestive heart failure, hypertension, age, diabetes mellitus, stroke, vascular disease, age, sex category). Patients with CTS usually present at an older age due to their lower risk of cyanosis and asymptomatic AF.

## Introduction and background

Cor triatriatum is a rare congenital cardiac condition characterized by the division of one atrium into two chambers by a fibromuscular membrane, resulting in three atrial chambers [1]. The incidence of cor triatriatum ranges from 0.1% to 0.4% in patients with clinically diagnosed cardiomyopathy and congenital heart disease [2]. The male-to-female ratio is 1.5:1, with no genetic association [3]. This abnormality is labeled cor triatriatum sinister (CTS) when the left atrium is involved and cor triatriatum dexter (CTD) when the right atrium is involved. In CTS, the posterosuperior chamber receives blood from four pulmonary veins, whereas the anteroinferior chamber communicates with the left ventricle through the mitral valve [3,4]. CTD, however, is caused by the persistence of the right valve of the right horn of the sinus venosus [5]. The presentations and complications of cor triatriatum vary widely. This study sought to determine the associations of cor triatriatum with cyanosis, atrial fibrillation (AF), and stroke.

## Review

Search strategy and selection criteria

MEDLINE (PubMed, Ovid), Embase, and Cochrane databases were searched for relevant articles on April 25, 2019. There were no language or time restrictions. The search strategies included various combinations of text words such as ‘triatriatum dextrum', ‘triatriatum sinistrum', ‘three atria', and ‘atrial membrane’ and medical subject headings such as ‘cor triatriatum’, ‘cor triatriatum dextrum'. and ‘cor triatriatum sinistrum'. In addition, references cited by all selected articles were searched manually to identify studies that were missed by the initial search.

Studies were included if they recruited subjects with any type of cor triatriatum and described the presentation, diagnosis, complications, and outcomes of cor triatriatum. Studies with insufficient data, posters, and conference papers were excluded, as were studies with insufficient descriptions of their subjects.

Study selection

Selected article titles and abstracts were reviewed independently by three authors (HR, MS, and SR), with the articles selected as meeting the inclusion criteria reviewed by a fourth author (WU). Potentially relevant full-text articles were also reviewed by all four authors to confirm their eligibility. Disagreements were resolved by mutual consensus and after detailed group discussions.

Data abstraction and analysis

**Figure 1:**
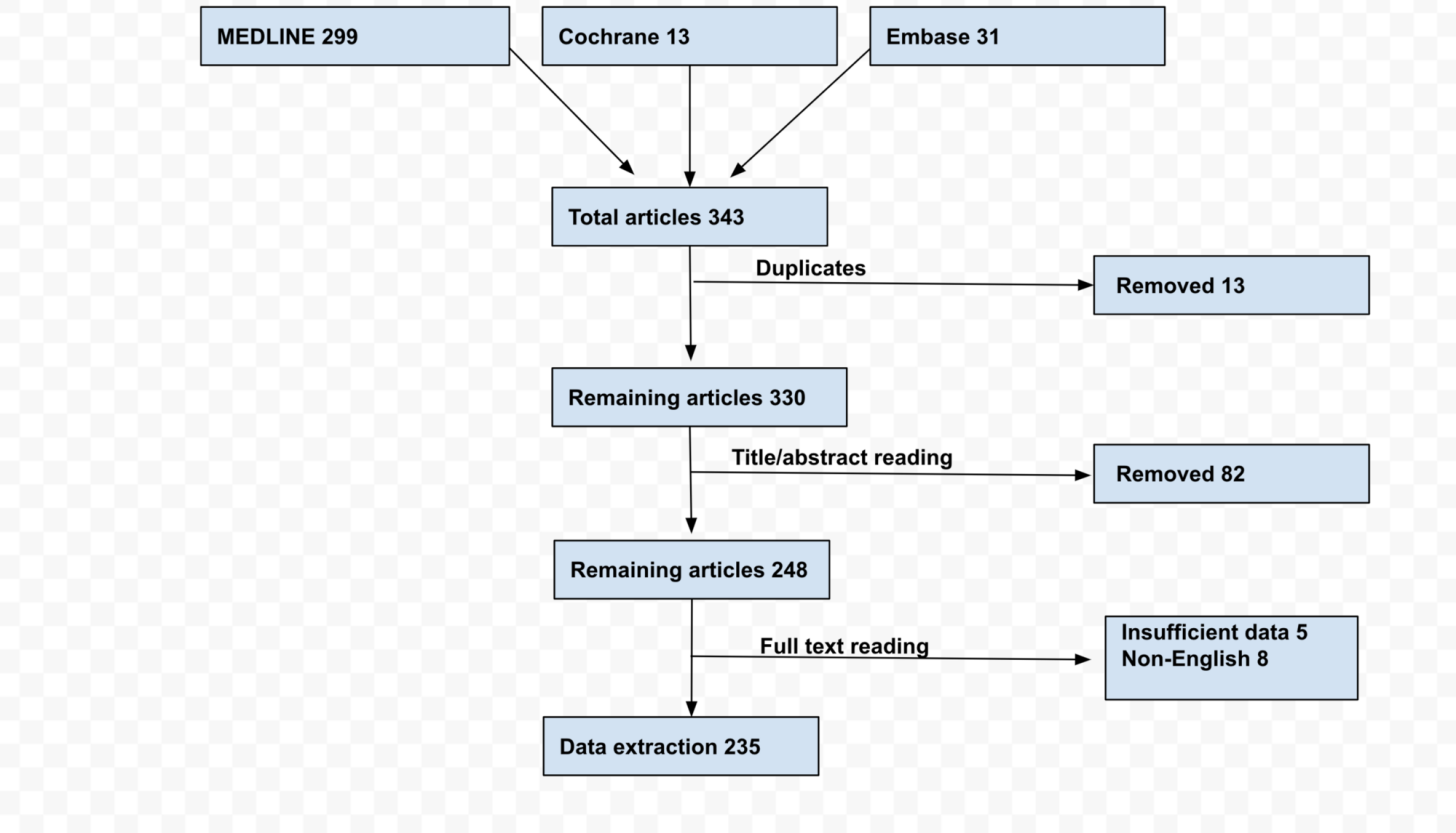
PRISMA flowsheet of selected articles on cor triatriatum CTS, cor triatriatum sinister; CTD, cor triatriatum dexter.

Following initial screening and removal of duplicates, 235 articles were selected using PRISMA (Preferred Reporting Items for Systematic Reviews and Meta-Analyses) guidelines (Figure 1).

Data from each article were extracted by two authors (HR and MS), including the types and cor triatriatum, the presentation of patients with this condition, diagnostic echocardiographic findings, patient management, and patient outcomes. Categorical data were dichotomized; nominal and ordinal data were reported as frequencies and proportions, respectively; and continuous data were reported as means and standard deviations. Categorical data were analyzed by the Pearson chi-squared test, with the alpha criterion for significance set at <0.05, but only used if the expected count in more than 20% of cells was >5. Otherwise, likelihood ratios were analyzed. All statistical analyses were performed using IBM SPSS Statistics for Windows, Version 22.0 (IBM Corp., Armonk, NY).

A review of the selected studies identified 191 patients with cor triatriatum, 103 (54%) males and 88 (46%) females, with a mean age of 29±23 years. Approximately 83% of patients with cor triatriatum had CTS and 17% had CTD. Mean ages at diagnosis differed significantly in patients with CTS and CTD (31±23 years vs. 21±20 years, p=0.02). CTS showed a significantly greater association with AF (14.65% vs. 12.5%, p=0.036) and had a substantially higher risk of stroke (7.9% vs. 5.0%, p=0.04) than CTD. In contrast, CTD was associated with a significantly higher rate of cyanosis than CTS (5.46% vs. 5.32%, p=0.05).

Of the patients with CTS and CTD, 13% and 12%, respectively, were diagnosed incidentally (p=0.24). Other frequent presentations of CTS and CTD that did not differ significantly in these two groups included atrial septal defect (ASD: 15.13% vs. 15.6%, p=0.89), dyspnea (29.60% vs. 29.32%, p=0.307), arrhythmia (5.99% vs. 6.25%, p=0.117), chest pain (4.9% vs. 5%, p=0.69), palpitations (4.9% vs. 5%, p=0.15), syncope (3.95% vs. 3.75%, p=0.216), hemoptysis (2.63% vs. 3.12%, p=0.521), congestive heart failure (3.75% vs. 3.75%, p=0.436), and pulmonary hypertension (1.64% vs. 1.56%, p=0.423; Table 1)

**Table 1:** Frequencies of symptoms in patients with CTS and CTD CTS, cor triatriatum sinister; CTD, cor triatriatum dexter.

Presentation	CTS	CTD	P-value
Dyspnea	29.60%	29.32%	p=0.307
Incidental finding	12.5%	12.5%	p=0.24
Chest pain	4.9%	5%	p= 0.69
Palpitations	4.9%	5%	p=0.15
Syncope	3.95%	3.75%	p=0.216
Hemoptysis	2.63%	3.12%	p=0.521
Arrhythmia	5.99%	6.25%	p=0.117
Cyanosis	5.46%	5.32%	p=0.05
Congestive heart failure	3.75%	3.75%	p=0.436
Pulmonary hypertension	1.64%	1.56%	p=0.423
Diagnosed at autopsy	2.70%	2.81%	p=0.176

 

Management did not differ significantly in patients with CTS and CTD (p=0.29) and included surgery (68.51% vs. 68.96%), medical management (12.23% vs. 12.06%), observation (12.79% vs. 12.06%), and balloon septoplasty/septostomy (6.29% vs. 6.89%; Table 2).

**Table 2:** Management strategies in patients with CTD and CTS CTS, cor triatriatum sinister; CTD, cor triatriatum dexter.

	CTS	CTD
Surgery	67.8%	72.4%
Observation	14.0%	6.9%
Medication	13.3%	6.9%
Balloon septoplasty	2.1%	6.9%
Balloon septostomy	2.8%	6.9%

The overall mortality rate was 16%; mortality rates did not differ significantly among the different management groups (p=0.33) or between patients with CTS and CTD (p=0.9).

Cor triatriatum history and clinical data

Cor triatriatum, also known as triatrial heart, was initially described in 1868. Cor triatriatum is a rare congenital cardiac anomaly in which a fibromuscular membrane divides atrium into two chambers, separating the pulmonary veins from the mitral valve and the vena cava from the tricuspid valve [6]. A septum in the left atrium results in CLS, whereas a septum in the right atrium results in CTD.

Cor triatriatum has also been classified based on size and fenestrations in the septa, with the first category having no opening, the second having one or more openings, and the third having a wide opening [3]. Embryologic malseptation, or entrapment, is thought to result in cor triatriatum [3,7]. The clinical presentation of cor triatriatum depends on the size and number of fenestrations, with a large single fenestration being asymptomatic whereas narrow openings present earlier as respiratory distress [8]. The presence of an atrial shunt is also associated with the severity of presentation and classifies cor triatriatum into different groups and classes [9]. Cor triatriatum has been classified into five categories based on morphology [10] and into diaphragmatic, hourglass, or tubular cor triatriatum based on the shape of the accessory left atrial chamber [11].

This study reviewed the presentation, management, and outcomes of both CTS and CTD. The symptomatic presentation of cor triatriatum varies with age. Cor triatriatum usually presents during infancy or childhood but can present at a very early age or later in life [12]. The mean±SD age of cor triatriatum patients in this study was 29±23 years. Cor triatriatum has a slight male predominance [6]. The onset, severity, and outcomes of presentation depend on the size of the communications between chambers and the presence of an ASD and associated cardiac lesions. The presentation of cor triatriatum in adults can range from an incidental finding to life-threatening hemoptysis, stroke, or heart failure [13-15]. Infants, however, can present with poor feeding, growth retardation, and/or failure to thrive [16,17].

CTS was found to be associated with substantially higher rates of AF and stroke than CTD. The left atrium is the origin of most AFs, and an additional septum in the left atrium can potentially increase the chances of AF [18]. AF increases the risk of clot formation in the left atrium causing many thromboembolic events. Moreover, the combination of AF and ASD is associated with a greater risk of strokes due to paradoxical embolization. The higher risk of stroke in CTS than in CTD patients may also be due to the additional membrane in the left atrium of the former, making the blood flow more turbulent. It is unclear whether surgical closure of the ASD and removal of the extra membrane would reduce the risk of stroke. It is imperative to address AF in these patients, irrespective of their CHA₂DS₂-VASc scores (congestive heart failure, hypertension, age, diabetes mellitus, stroke, vascular disease, age, sex category) due to their higher risks of thromboembolism.

The diagnosis of cor triatriatum depends on multiple imaging techniques, with echocardiography being the diagnostic modality of choice. Newer three-dimensional echocardiography can reveal the exact location of the defect, as well as chamber size, septal defects, and whether the patient has valvular or chamber stenosis, thereby helping to guide the surgical approach. Pulse wave Doppler technologies can detect the maximum and mean pressure differences across the interatrial membranes and chambers [19]. More than 80% of our patients were diagnosed with echocardiography. In selected patients, computed tomography and magnetic resonance imaging can be used to show turbulent flow across the interatrial membrane [20-22]. Cardiac catheterization can also help in the assessment of pressure and resistance associated with the ventricular obstruction [23], and chest X-ray findings can suggest pulmonary venous congestion and atrial dilation. An electrocardiogram is useful for showing P wave morphology consistent with atrial enlargement.

CTS and CTD are best managed by surgery. It is crucial to delineate the complex anatomy of cor triatriatum in each patient before surgery. In CTS, long-standing inflow obstruction of the left ventricle can increase the risks of backflow pulmonary venous congestion and pulmonary artery hypertension. The reversibility of pulmonary congestion should be ensured before surgery in patients with CTS [24]. Pulmonary congestion and pulmonary arterial hypertension are never present in patients with CTD. However, pulmonary blood flow in patients with CTD is reduced by the obstruction of flow in the right ventricle that can worsen cyanosis. Therefore, transesophageal echocardiography-guided volume administration is crucial to maintain pulmonary flow in patients with CTD [25-27]. Other management options, including balloon septoplasty, balloon septostomy, and observation, can act as a bridge to surgery [24,28]. Generally, medical management has no role in the treatment of CTS and CTD, but it is unclear if CTS patients require anticoagulation to prevent stroke.

Patient survival outcomes depend on presentation and management. Early surgical management offers better survival outcomes [26,27]. For example, one study of 25 patients with cor triatriatum who underwent surgery at age 19 years showed a 10-year survival rate of 83% [28]. In our analysis, 69% of patients were managed by cardiovascular surgeons, with these patients having an overall survival rate of 84%.

Limitations

We were unable to perform a meta-analysis of the data because there are no large-scale studies of patients with this rare condition, with most of the data obtained from individual case reports. This study, therefore, assessed the temporal relationships of different variables and the associations of CTS and CTD with various factors. Large-scale studies are needed to determine the causes of CTS and CTD and to determine the safety of anticoagulation therapy in patients with CTS.

## Conclusions

The goal of the study was to compare the presentation, diagnosis, and management of CTS and CTD by reviewing the relevant cases available in the literature. Our results showed that AF, ASD, and stroke are more prevalent in CTS than CTD. Therefore, early anticoagulation and surgical management of patients with asymptomatic or symptomatic CTS will lead to better clinical outcomes.
